# Center of Mass Offset Enhances the Selection of Transverse Gallop in High-Speed Running by Horses: A Modeling Study

**DOI:** 10.3389/fbioe.2022.825157

**Published:** 2022-02-28

**Authors:** Takumi Yamada, Shinya Aoi, Mau Adachi, Tomoya Kamimura, Yasuo Higurashi, Naomi Wada, Kazuo Tsuchiya, Fumitoshi Matsuno

**Affiliations:** ^1^ Department of Mechanical Engineering and Science, Graduate School of Engineering, Kyoto University, Kyoto, Japan; ^2^ Department of Aeronautics and Astronautics, Graduate School of Engineering, Kyoto University, Kyoto, Japan; ^3^ Department of Electrical and Mechanical Engineering, Nagoya Institute of Technology, Nagoya, Japan; ^4^ Laboratory of System Physiology, Joint Faculty of Veterinary Medicine, Yamaguchi University, Yamaguchi, Japan

**Keywords:** horse, transverse gallop, center of mass offset, gait selection, model

## Abstract

Horses use the transverse gallop in high-speed running. However, different animals use different gaits, and the gait preference of horses remains largely unclear. Horses have fore-aft asymmetry in their body structure and their center of mass (CoM) is anteriorly located far from the center of the body. Since such a CoM offset affects the running dynamics, we hypothesize that the CoM offset of horses is important in gait selection. In order to verify our hypothesis and clarify the gait selection mechanisms by horses from a dynamic viewpoint, we developed a simple model with CoM offset and investigated its effects on running. Specifically, we numerically obtained periodic solutions and classified these solutions into six types of gaits, including the transverse gallop, based on the footfall pattern. Our results show that the transverse gallop is optimal when the CoM offset is located at the position estimated in horses. Our findings provide useful insight into the gait selection mechanisms in high-speed running of horses.

## 1 Introduction

Horses use the transverse gallop in high-speed locomotion. This gait has one flight phase in one gait cycle. Specifically, the hind legs first touch the ground, and then the fore legs touch the ground. After that, a flight phase appears. This gait is different from the rotary gallop in cheetahs, which has two flight phases, each of which appears after the touchdowns of the fore legs and those of the hind legs (Bertram and Gutmann, 2008; Biancardi and Minetti, 2012). The gaits of quadrupeds when running at their fastest speeds vary between species, and it remains unclear why horses use the transverse gallop.

Horses have fore-aft asymmetry in their body structure. In particular, they have a long neck, and their center of mass (CoM) is anteriorly located and far from the center of the body (Buchner et al., 1997; Self Davies et al., 2019). Such a CoM offset affects the dynamics of the running motion. For example, when the fore-aft CoM location of dogs was changed by carrying a weight during trotting, which is characterized by the simultaneous touchdown of the diagonal fore and hind legs, the footfall pattern changed (Lee et al., 2004). Specifically, the fore and hind legs came to touch the ground first when the load was applied to the anterior and posterior sides, respectively. In other words, the CoM offset changed the gait. Therefore, we hypothesize that the CoM offset of horses plays an important role in their gait selection.

Since animal locomotion is a complex phenomenon generated through dynamical interactions between the body mechanical system, the nervous system, and the environment, it is difficult to fully understand the mechanisms for gait selection in animals only from observation. Therefore, simple models, which extract essential elements for the running dynamics, have been used to clarify the mechanisms (Tanase et al., 2015; Gan et al., 2016; Chen et al., 2019; Kamimura et al., 2021). Poulakakis et al. (2006) used a simple quadrupedal model and showed the relationship between the pitch angular velocity and the number of flight phases in one gait cycle during bounding gait. In addition, Zou and Schmiedeler (2006) used a model focusing on the vertical and pitch movements and derived a stability condition depending on the CoM offset. However, the model did not incorporate horizontal movement, and the mechanism for the gait selection remains unclear.

In the present study, we investigated the effects of the CoM offset on quadrupedal running in order to verify our hypothesis from a dynamic viewpoint. Specifically, we constructed a bounding model with CoM offset and searched periodic solutions by numerical simulations. We then classified the obtained solutions into six types of gaits depending on the footfall pattern and examined which gait is optimal based on performance criteria. Our findings provide useful insight into the mechanisms for high-speed running in horses.

## 2 Methods

### 2.1 Model

**FIGURE 1:**
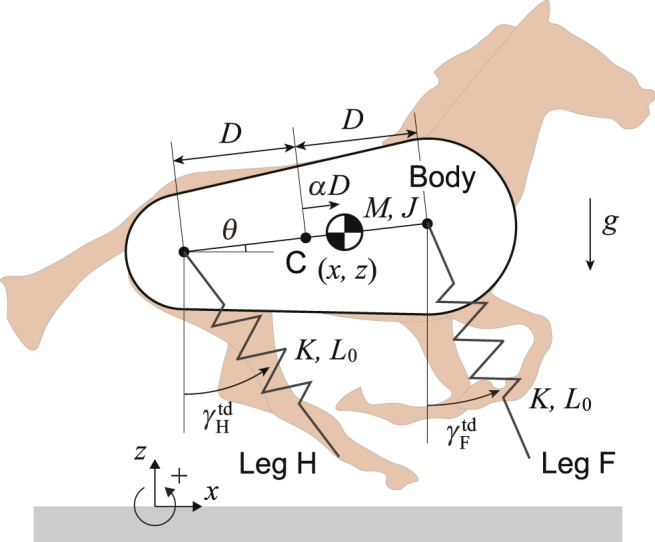
Horse model composed of a rigid body with center of mass offset and two massless springs.

We used a horse model composed of a rigid body and two massless springs on the sagittal plane (Figure 1). The springs represent the fore and hind legs (Legs F and H) and are connected to the body by smoothly rotating joints. Here, *M* and *J* are the mass and moment of inertia around the CoM of the body, respectively. The distance between the leg joints is 2*D*. The CoM is located at a distance of *αD* (−1 ≤ *α* ≤ 1) from the center C between the leg joints, where *α* = 0 corresponds to C, and *α* = 1 and −1 correspond to the joints of the fore and hind legs, respectively. Moreover, *x* and *z* are the horizontal and vertical positions of the CoM, respectively, and *θ* is the pitch angle relative to the horizontal line. The spring constant and neutral length of both the fore and hind legs are *K* and *L*_0_, respectively. When Leg *i* (*i* = F, H) is in the air, its length remains *L*_0_ and its angle also maintains the touchdown angle $\gamma_{i}^{\text{td}}$ . The positive direction of these angles is counterclockwise. When the tip of the leg reaches the ground, it is constrained to the ground and behaves as a frictionless pin joint. When the leg length returns to *L*_0_ after the compression, the tip leaves the ground. Since the touchdown and liftoff occur at the neutral length and our model has no dissipative component, such as friction or a damper, our model is energy conservative.

The equations of motion of the model are given by

$$M\ddot{x}=\underset{i=F,H}{\sum }-F_{i}\sin\gamma_{i}$$ (1a)

$$M\ddot{z}=\underset{i=F,H}{\sum }F_{i}\cos\gamma_{i}-Mg$$ (1b)

$$J\ddot{\theta }=F_{F}\left(1-\alpha \right)D\cos\left(\gamma_{F}-\theta \right)-F_{H}\left(1+\alpha \right)D\cos\left(\gamma_{H}-\theta \right),$$ (1c)

where

$$F_{i}=\left\{\begin{array}{cc} 0\; & \;swingphase\\ K\left(L_{0}-L_{i}\right)\; & \;stancephase \end{array}\right.\;i=F,H$$ (2)

and *L*_*i*_ and *γ*_*i*_ (*i* = F, H) are the length and angle, respectively, of Leg *i* relative to the vertical line. Moreover, *γ*_*i*_ is determined by the joint and touchdown positions of Leg *i*. The touchdown condition $r_{i}^{\text{td}}=0$ and liftoff condition $r_{i}^{\text{lo}}=0$ of Leg *i* are given by

$$\begin{array}{c} r_{i}^{\text{td}}=z+\varepsilon_{i}-\alpha D\sin\theta -L_{0}\cos\gamma_{i}^{\text{td}}=0\\ r_{i}^{\text{lo}}=L_{0}-L_{i}=0, \end{array}\;i=F,H$$ (3)

where *ɛ*_F_ = 1 and *ɛ*_H_ = −1.

The physical parameters of the model were determined based on the estimated values of Thoroughbreds (*Equus ferus caballus*). In particular, we used *M* = 490 kg and *J* = 167 kgm^2^ based on Swanstrom et al. (2005). We used *D* = 0.48 m from the distance between the shoulder and hip joints and *L*_0_ = 1.33 m from the average value of the distances between the shoulder joint and the toe of the fore limb and between the hip joint and the toe of the hind limb based on Grossi and Canals, (2010). We used *K* = 45.4 kN/m based on Farley et al. (1993).

### 2.2 Gait

**FIGURE 2:**
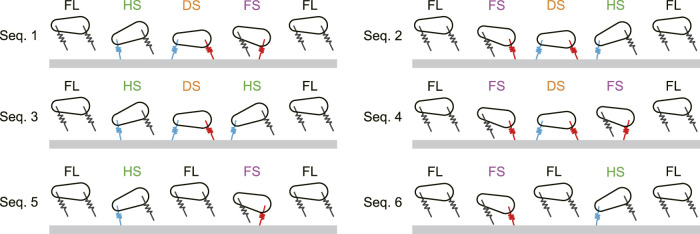
Six possible phase transitions (Sequences 1 … 6) from one apex to the next apex. FL, FS, HS, and DS stand for flight, fore leg stance, hind leg stance, and double stance, respectively.

The gait is generally determined based on the order of touchdown and liftoff of the legs. We defined the following four phases: flight (FL), fore leg stance (FS), hind leg stance (HS), and double stance (DS). In FL, both legs are in the air. In FS, only the fore leg is in contact with the ground. In HS, only the hind leg is in contact with the ground. In DS, both legs are in contact with the ground. We investigated motions (periodic solutions) starting from an apex (i.e., $\dot{z}=0$ in FL) and returning to the next apex after each leg touches the ground once. The periodic solutions are obtained by the transitions between these phases. The phase transitions of the periodic solutions are classified into six sequences (Sequences 1 ... 6), as shown in Figure 2. In Sequence 1, the hind leg first touches the ground (HS), and then the fore leg touches the ground so that two legs are in contact with the ground (DS). After that, the hind leg first leaves the ground (FS), and then the fore leg leaves the ground to return to FL. This gait has one flight phase and one double stance phase and corresponds to the transverse gallop in horses (Hildebrand, 1977; Biancardi and Minetti, 2012). Sequence 2 is obtained by swapping the behaviors of the fore and hind legs in Sequence 1. In Sequence 3, the hind leg first touches the ground (HS), and then the fore leg touches the ground, so that two legs are in contact with the ground (DS). After that, the fore leg first leaves the ground (HS), and then the hind leg leaves the ground to return to FL. This gait also has one flight phase and one double stance phase. Sequence 4 is obtained by swapping the behaviors of the fore and hind legs in Sequence 3. In Sequence 5, the hind leg touches the ground (HS) and then leaves the ground to return to FL. After that, the fore leg touches the ground (FS), and then leaves the ground to once again return to FL. This gait has two flight phases but no double stance phase. Sequence 6 is obtained by swapping the behaviors of the fore and hind legs in Sequence 5. Sequences 5 and 6 are identical when the time profile of one sequence is shifted by half a gait cycle.

### 2.3 Search of Solutions

In order to find periodic solutions, we defined the Poincaré section at the apex of the CoM $\left(\dot{z}=0\right)$ . Since *x* monotonically increases during locomotion and is not periodic, we used $q={\left[z\;\theta \;\dot{x}\;\dot{\theta }\right]}^{⊤}$ as the state on the Poincaré section. We used the touchdown angles as the parameter set $u={\left[\gamma_{H}^{\text{td}}\;\gamma_{F}^{\text{td}}\right]}^{⊤}$ . The Poincaré map *P* is then defined as

$$q_{n+1}=P\left(q_{n},u_{n}\right)$$ (4)

where *q*_*n*_ is the state at the *n*th intersection with the Poincaré section, and *u*_*n*_ is the *n*th parameter set. A periodic solution satisfies

$$q^{*}=P\left(q^{*},u^{*}\right)$$ (5)

where *q* ^∗^ is the fixed point on the Poincaré section. We numerically searched fixed points for periodic solutions using the Newton-Raphson method.

### 2.4 Performance Criteria

In order to evaluate the obtained solutions, we used the gait stability as a performance criterion (Poulakakis et al., 2006; Tanase et al., 2015; Kamimura et al., 2021). In order to analyze the gait stability, we investigated the eigenvalues of the linearized Poincaré map around the fixed points on the Poincaré section. Since our model is energy conservative, the solution is asymptotically stable, when all of the eigenvalues, except for one eigenvalue of 1, are located inside the unit circle on the complex plane. Otherwise, the solution is unstable.

Horses stabilize their gaze during running by preventing the pitch movement of the body from disturbing the head (Dunbar et al., 2008). Therefore, we also used the fluctuation of the pitch movement of the body as another performance criterion, which is obtained from the difference between the maximum and minimum values of *θ* for one gait cycle.

## 3 Results

### 3.1 Effect of Center of Mass Offset on Gait Pattern

**FIGURE 3:**
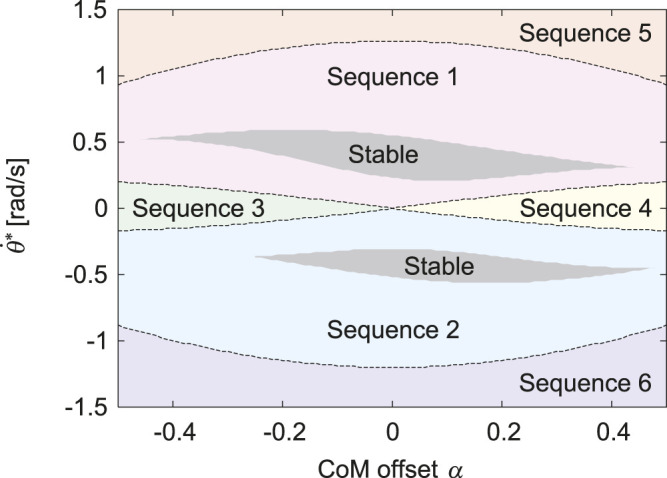
Gait classification of obtained periodic solutions for *α* and ${\dot{\theta }}^{*}$ . Gray regions indicate stable solutions.

First, we set the total energy of our model as *E* = 20.3 kJ (gravitational potential energy is 0 at the ground level) and the forward speed at the apex as ${\dot{x}}^{*}=7.5\;m/\text{s}$ (horizontal kinetic energy $T^{*}=M{\left({\dot{x}}^{*}\right)}^{2}/2=13.8\;kJ$ ) based on the measured data in horses (Minetti et al., 1999). We then searched periodic solutions in the range of −0.5 ≤ *α* ≤ 0.5 and $-1.5\leq {\dot{\theta }}^{*}\leq 1.5$ . As a result, we found a unique solution for each set of $\left(\alpha ,{\dot{\theta }}^{*}\right)$ in this range, the gait of which is classified into Sequences 1 through 6, as shown in Figure 3. The gait boundaries are symmetric with respect to *α* = 0 and ${\dot{\theta }}^{*}=0$ , and four boundaries of Sequences 1, 2, 3, and 4 meet at *α* = 0 and ${\dot{\theta }}^{*}=0$ . When *α* = 0, the solutions have four types of gait, labeled as Sequences 1, 2, 5, and 6. For the solutions with ${\dot{\theta }}^{*}>0$ , the hind leg first touches the ground. Specifically, Sequence 1 appears when ${\dot{\theta }}^{*}$ is small, and Sequence 5 appears when ${\dot{\theta }}^{*}$ is large. In contrast, for the solutions with ${\dot{\theta }}^{*}<0$ , the fore leg first touches the ground. Specifically, Sequence 2 appears when $\left|{\dot{\theta }}^{*}\right|$ is small, and Sequence 6 appears when $\left|{\dot{\theta }}^{*}\right|$ is large. In addition to the four gaits, Sequences 3 and 4 appear around ${\dot{\theta }}^{*}=0$ when *α* < 0 and when *α* > 0, respectively. As |*α*| increases, the range of ${\dot{\theta }}^{*}$ of Sequences 1 and 2 decreases and that of Sequences 3, 4, 5, and 6 increases. Stable solutions exist only in Sequences 1 and 2 at −0.46 < *α* < 0.48. Specifically, only Sequence 1 is stable when the CoM is located posteriorly at −0.46 < *α* < −0.25, and only Sequence 2 is stable when the CoM is located anteriorly at 0.44 < *α* < 0.48.

**FIGURE 4:**
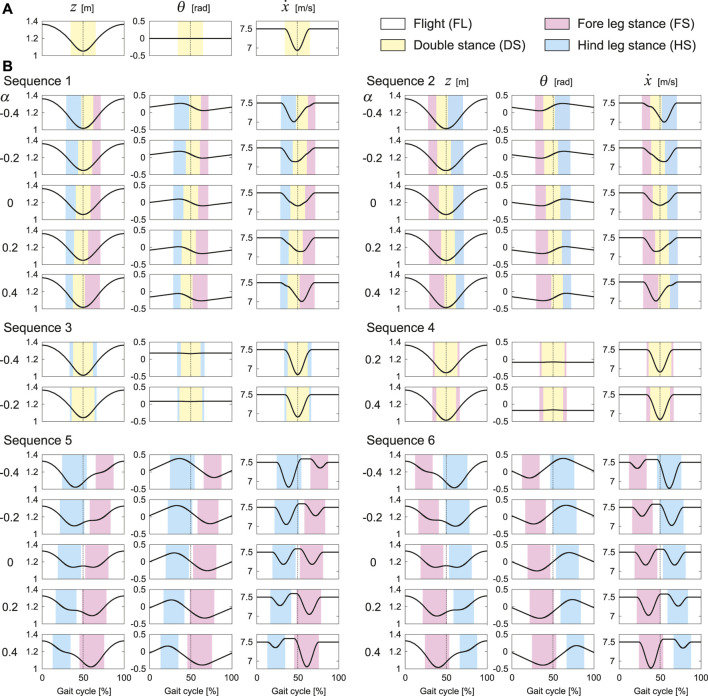
Characteristics of time profiles of $z,\theta ,and\dot{x}$ and phases of periodic solutions depending on *α* and gait. **(A)** Solution for *α* = 0 and ${\dot{\theta }}^{*}=0$ . **(B)** Typical solutions of each gait for *α* = 0, ±0.2, and ±0.4, where ${\dot{\theta }}^{*}=0.5,-0.5,0,0,1.5$ , and −1.5 rad/s are used for Sequences 1, 2, 3, 4, 5, and 6, respectively (see Supplementary Video S1).

Next, we investigate the time profiles of the periodic solutions in order to clarify the characteristition with *α*. First, Figure 4A shows the tixme profile of $z,\theta ,\;and\;\dot{x}$ of the solution for *α* = 0 and ${\dot{\theta }}^{*}=0$ , at which the four boundaries of Sequences 1, 2, 3, and 4 meet (Figure 3). In this case, the fore and hind legs touch and leave the ground simultaneously. The trajectories of *z* and $\dot{x}$ are symmetric with respect to 50% of the gait cycle, and *θ* is always zero. Next, Figure 4B shows the time profiles of typical solutions of each gait for *α* = 0, ±0.2, and ±0.4, where ${\dot{\theta }}^{*}=0.5,-0.5,0,0,1.5$ , and −1.5 rad/s are used for Sequences 1, 2, 3, 4, 5, and 6, respectively. As a common feature of all gaits, when *α* = 0, the timings of touchdown and liftoff are shifted depending on ${\dot{\theta }}^{*}$ and are no longer simultaneous between the fore and hind legs. However, the trajectories of *z* and $\dot{x}$ remain symmetric with respect to 50% of the gait cycle, regardless of ${\dot{\theta }}^{*}$ . Although *θ* ^∗^ remains 0, specific waveforms appear in *θ* depending on ${\dot{\theta }}^{*}$ . The trajectory of *θ* is symmetric with respect to the intersection of *θ* = 0 and 50% of the gait cycle. As *α* increases, the stance phase duration increases and decreases for the fore and hind legs, respectively, and the trajectories and phases become asymmetric.

Sequences 1 and 2 have solutions for all *α* = 0, ±0.2, and ±0.4, the trajectories and phases of which are symmetric with respect to 50% of the gait cycle. As *α* increases, the onset and end of the DS phase are advanced in Sequence 1 and delayed in Sequence 2, whereas those of the FL phase remains almost unchanged. Here, *z* has a one-peak shape and remains almost unchanged. Although the waveform of *θ* remains almost unchanged, the mean value decreases. The timing at which $\dot{x}$ takes the minimum value is delayed in Sequence 1 and advanced in Sequence 2. However, the minimum value decreases as |*α*| increases.

Sequence 3 has solutions only for *α* = −0.2 and −0.4, and Sequence 4 has solutions only for *α* = 0.2 and 0.4. These trajectories are identical for the same |*α*|. In addition, these phases are also identical when the timings of touchdown and liftoff are swapped between the fore and hind legs. Unlike Sequences 1 and 2, the trajectories and the timings of touchdown and liftoff are symmetric with respect to 50% of the gait cycle regardless of *α*. Here, *z* has a one-peak shape and remains almost unchanged as *α* increases. The waveform of *θ* remains almost unchanged, whereas the mean value decreases. The minimum value of $\dot{x}$ decreases as |*α*| increases.

Sequences 5 and 6 have solutions for all *α* = 0, ±0.2, and ±0.4, the trajectories and phases of which are symmetric with respect to 50% of the gait cycle. When *α* = 0, *z* has a two-peak shape. As |*α*| increases, one of the two peaks decreases and the two-peak shape changes into a one-peak shape. As *α* increases, the FS and HS phases are advanced in Sequence 5 and delayed in Sequence 6. Whereas the mean value of *θ* remain almost unchanged, the peak timings change in accordance with changes in the FS and HS phases. Since Sequences 5 and 6 have two FL phases, $\dot{x}$ has two minimum values in the FS and HS phases. Regardless of *α*, our model is accelerated in the HS phase and decelerated in the FS phase in Sequence 5, and vice versa in Sequence 6. As *α* increases, the minimum value of $\dot{x}$ in the HS phase increases and that in the FS phase decreases in both Sequences 5 and 6.

### 3.2 Effect of Speed on Gait Performance

Although the previous section investigated the effects of the CoM offset *α* on the gait pattern using the average speed, horses have a wide range of speed for galloping (Hoyt and Taylor, 1981; Minetti et al., 1999). In this section, we investigate the effects of speed on the gait characteristics using the estimated value of *α* in horses (*α* = 0.2) (Self Davies et al., 2019) compared with those using *α* = 0.

**FIGURE 5:**
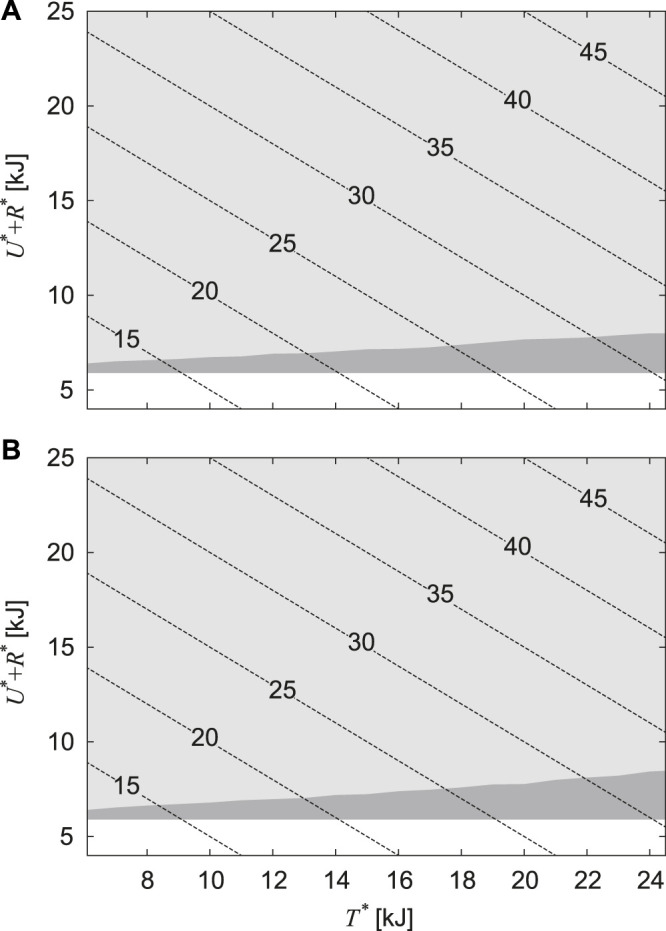
Regions in which periodic solutions are found for horizontal translational kinetic energy T* and the sum of gravitational potential energy and rotational kinetic energy U* + R*. **(A)** *α* = 0.2 and **(B)** *α* = 0. The light and dark gray regions indicate that the solutions are unstable and stable, respectively. The dotted lines indicate the contour of total energy *E*.

The total energy *E* of our model is explained by the horizontal translational kinetic energy $T^{*}=M{\left({\dot{x}}^{*}\right)}^{2}/2$ , gravitational potential energy *U* ^∗ ^= *Mgz* ^∗^, and rotational kinetic energy $R^{*}=J{\left({\dot{\theta }}^{*}\right)}^{2}/2$ at the apex (*E* = *T* ^∗^ + *U* ^∗ ^+ *R* ^∗^). We searched for periodic solutions by changing *T* ^∗^ and *U* ^∗^ + *R* ^∗^ from the previous results of *α* = 0.2 and 0. Figures 5A,B compare the region for *T* ^∗^ and *U* ^∗^ + *R* ^∗^ where periodic solutions are found and that where stable periodic solutions are found for *α* = 0.2 and 0, respectively. In both figures, although solutions, including unstable solutions, are widely distributed for *T* ^∗^ and *U* ^∗ ^+ *R* ^∗^ (no solution is found below 5.9 kJ of *U* ^∗ ^+ *R* ^∗^), stable solutions exist only in a limited range for *U* ^∗^ + *R* ^∗^. In other words, when the forward speed increases, only the horizontal translational kinetic energy increases, whereas the other energies are almost unchanged in the stable solutions.

**FIGURE 6:**
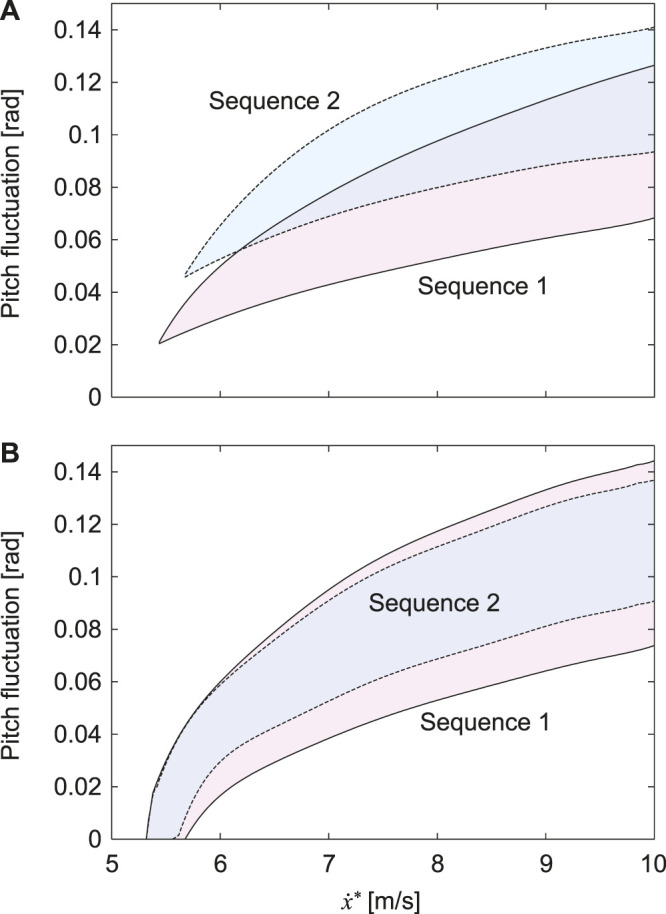
Fluctuation of pitch movement of stable solutions for ${\dot{x}}^{*}$ . **(A)** *α* = 0.2 and **(B)** *α* = 0.

Next, we searched for periodic solutions by using *U* ^∗^ + *R* ^∗^ = 6.6 kJ, which corresponds to the value obtained from *E* = 20.3 kJ and *T* ^∗^ = 13.8 kJ used in Figure 3, and by changing ${\dot{x}}^{*}$ in 5–10 m/s of the speed range (*T* ^∗^ = 6.1–24.5 kJ) of the horse galloping (Hoyt and Taylor, 1981; Minetti et al., 1999). Figures 6A,B show the fluctuation of the pitch movement of the body for ${\dot{x}}^{*}$ of the obtained stable solutions for *α* = 0.2 and 0, respectively. In both figures, only Sequences 1 and 2 have stable solutions in the same way as Figure 3. When *α* = 0.2, the stable solutions of Sequence 1 exist in a wider range of ${\dot{x}}^{*}$ and have smaller pitch fluctuations than those of Sequence 2. In contrast, when *α* = 0, the range of ${\dot{x}}^{*}$ of the stable solutions is almost identical and the pitch fluctuations also are not much different between Sequences 1 and 2.

## 4 Discussion

### 4.1 Effect of Center of Mass Offset on Gait

The proposed model has six types of gaits, labeled Sequences 1 through 6 (Figure 2). In Sequences 1, 2, 5, and 6, when a leg first touches the ground, this leg leaves the ground earlier than the other leg. As a result of the search of periodic solutions, we found only these four sequences when the CoM is located at the center (*α* = 0), as shown in Figure 3. In Sequences 3 and 4, when a leg first touches the ground, this leg leaves the ground later than the other leg (Figure 2). Sequences 3 and 4 appeared only when the CoM is located posteriorly (*α* < 0) and anteriorly (*α* > 0), respectively (Figure 3). In other words, the introduction of the CoM offset *α* to the model allowed Sequences 3 and 4 to appear.

Whereas the trajectories and phases of the periodic solutions were symmetric for *α* = 0, the solutions became asymmetric as |*α*| increased (Figure 4). However, the asymmetric tendency depended on the gait. Specifically, as |*α*| increased, the trajectories and phases showed higher asymmetry in order of Sequences 3 and 4, Sequences 1 and 2, Sequences 5 and 6 (Figure 4). These reasons can be explained from the viewpoint of the dynamics of the body rotation. Specifically, the body rotation is created by the moment of force by the ground reaction forces from the fore and hind legs. Therefore, the periodic solutions require the moment of impulse generated for one gait cycle to be balanced between the fore and hind legs. When *α* > 0, the distances from the CoM to the joints of the fore and hind legs are short and long, respectively. Therefore, the moment of impulse is balanced by increasing the magnitude of the ground reaction force and stance phase duration of the fore leg and by decreasing those of the hind leg, and vice versa when *α* < 0. In the DS phase, the net moment applied to the body is reduced by the positive moment from the fore leg and negative moment from the hind leg, which decreases the asymmetry of the body rotation. Since the DS phase duration decreased in order of Sequences 3 and 4, Sequences 1 and 2, Sequences 5 and 6, the asymmetry of the trajectories and phases increased in this order.

This can also explain why Sequences 3 and 4 appear in *α* < 0 and *α* > 0, respectively. Specifically, in Sequences 3 and 4, when a leg first touches the ground, this leg leaves the ground later than the other leg (Figure 2). Based on the solution for *α* = 0 and ${\dot{\theta }}^{*}=0$ , where both legs touch and leave the ground simultaneously (Figure 4A). Since the stance phase duration of the hind leg became longer than that of the fore leg for *α* < 0, Sequence 3 appeared, and vice versa for *α* > 0 and Sequence 4 (Figure 4B).

### 4.2 Gait Selection by Horses

Sequence 1 has one flight phase, after which the hind leg first touches the ground (Figure 2), and thus corresponds to the transverse gallop used by horses and gnus (Muybridge, 1957; Pennycuick, 1975; Hildebrand, 1977; Hildebrand, 1989). Sequence 2 has one flight phase, after which the fore leg first touches the ground (Figure 2), and thus corresponds to the transverse gallop used by deer and antelopes (Bigalike, 1972; FitzGibbon and Fanshawe, 1988; Hildebrand, 1989). Sequences 3 and 4 also have one flight phase (Figure 2). When touchdown and liftoff occur almost simultaneously between the fore and hind legs and the pitch fluctuation is small, as obtained in Figure 4B, these gaits correspond to the pronk used by springboks and Thomson’s gazelles (Bigalike, 1972; FitzGibbon and Fanshawe, 1988; Hildebrand, 1989). In contrast, Sequences 5 and 6 have two flight phases and thus correspond to the rotary gallop used by cheetahs and greyhounds (Muybridge, 1957; Hildebrand, 1977; Hildebrand, 1989; Bertram and Gutmann, 2008; Biancardi and Minetti, 2012; Hudson et al., 2012).

When we used the physical parameters estimated in horses, including the CoM offset *α* = 0.2, only Sequences 1 and 2 had stable solutions (Figures 3, 6). Sequence 1 had a wider speed range (5.5–10 m/s) than Sequence 2 (5.8–10 m/s) (Figure 6) and the speed range of Sequence 1 was closer to that of a galloping horse (5–10 m/s) (Hoyt and Taylor, 1981; Minetti et al., 1999). Furthermore, Sequence 1 had smaller pitch fluctuations (2.2–7.2 deg) than Sequence 2 (3.2–7.9 deg) (Figure 6A), and the amount of the fluctuations of Sequence 1 was closer to that of a galloping horse (2–8 deg) (Dunbar et al., 2008). However, when we used *α* = 0 instead of *α* = 0.2 estimated in horses, although Sequences 1 and 2 also had stable solutions, the speed range and pitch fluctuation were not much different between Sequence 1 and 2 (Figure 6B). Our results suggest that Sequence 1, which corresponds to the transverse gallop actually used by horses, is a suitable gait for horses from a dynamical viewpoint.

### 4.3 Vertical and Pitch Movements

Although stable periodic solutions existed for a large range of the horizontal translational kinetic energy *T* ^∗^, these solutions existed for a limited range of the sum of the gravitational and rotational kinetic energies *U* ^∗ ^+ *R* ^∗^ (Figure 5). This means that the stability of bounding mainly depends on the vertical and pitch movements of the body. Thus far, simple models focusing on the vertical and pitching movements have been used to investigate the gait stability (Berkemeier, 1998; Zou and Schmiedeler, 2006; De and Koditschek, 2018; Kamimura et al., 2021). In particular, Berkemeier (1998) investigated the stability of bounding (which corresponds to Sequences 5 and 6) using a symmetrical model, which is identical to our model with *α* = 0, and derived the stability condition as *μ* < 1, where *μ* = *J*/(*MD* ^2^). Zou and Schmiedeler (2006) improved his model by introducing the CoM offset *α*, as in the present study, and derived the stability condition for *α* > 0 as *μ* < 1 − *α* ^2^. For the physical parameter *μ* = 1.48 estimated in horses (Swanstrom et al., 2005), our results showed that all solutions of Sequences 5 and 6 were unstable, regardless of *α* (Figure 3), which is consistent with their results.

While dogs have a larger CoM offset (*α* = 0.28) (Ben-Amotz et al., 2020) than horses (*α* = 0.2) (Self Davies et al., 2019), they use both transverse and rotary gallop depending on the speed (Biancardi and Minetti, 2012). Polet (2021) showed that the pitch moment of inertia plays an important role for the gait determination using a simple model. In addition to the CoM offset, we would like to investigate the contribution of the pitch moment of inertia to the gait selection in the future.

### 4.4 Limitations and Future Research

Ground reaction forces of animals during fast running show sinusoidal patterns (Alexander et al., 1986; Full and Tu, 1991; Farley et al., 1993). Blickhan (1989) and McMahon and Cheng (1990) introduced a simple spring-mass model to achieve these patterns for the ground reaction forces. This representation of the leg by a linear spring successfully described and predicted animal locomotion (Blickhan and Full, 1993; Farley et al., 1993; Deng et al., 2012; Tanase et al., 2015; Gan et al., 2016). For example, Gan et al. (2016) reproduced three different gaits (walk, trot, and tölt) of horses by using a quadrupedal spring-mass model and suggested that different quadrupedal gaits are interpreted as different elastic oscillations. Moreover, passively stable running allows the controller and sensing to be simple, even when there are disturbances (Poulakakis et al., 2006). Therefore, such a simple passive model is useful to investigate the gait selection mechanisms by animals (Tanase et al., 2015; Kamimura et al., 2021). However, actual animals lose kinetic energy by collisions of their legs with the ground and by dissipation via friction and compensate for this loss by their muscles. Energy efficiency is an important factor for animal gait (Ruina et al., 2005; Chatzakos and Papadopoulos, 2009; Cao and Poulakakis, 2015; Polet and Bertram, 2019; Polet, 2021). We would like to introduce the elements for energy dissipation and generation in order to obtain a deeper understanding of the running mechanism in animals in the future.

In addition to the CoM offset and pitch moment of inertia, different characteristics between the fore and hind legs could also influence the running dynamics. For example, the muscle mass of the hind legs is greater than that of the fore legs in horses, and it has been suggested that the main role of the fore legs is to support the body weight, whereas that of the hind legs is to generate driving forces (Payne et al., 2005; Crook et al., 2008). Therefore, future investigations of the effects of different characteristics of the legs would be useful for a better understanding of the relationship between the body structure and running in animals.

In the present study, we used the physical parameters estimated in horses to discuss the gait selection by horses. Physical parameters, such as body weight, moment of inertia, and leg length, vary between species. Different parameters could influence the gait preference. We would like to investigate gait selection by animals other than horses in order to clarify the mechanisms for difference gaits between species in future studies.

## Data Availability

The raw data supporting the conclusion of this article will be made available by the authors, without undue reservation.
